# Radiation-Associated Angiosarcoma of the Breast: The State of the Art of a Rare and Aggressive Disease

**DOI:** 10.3390/jpm14080859

**Published:** 2024-08-14

**Authors:** Salvatore Cozzi, Sebastiano Finocchi Ghersi, Francesca Tava, Lilia Bardoscia, Masoumeh Najafi, Maria Paola Ruggieri, Anne-Agathe Serre, Camille Roukoz, Cristina Gutierrez Miguelez, Amina Lazrek, Angela Sardaro, Cecilia Taverna

**Affiliations:** 1 Radiation Oncology Department, Centre Léon Bérard, 69373 Lyon, France; agathe.serre@lyon.unicancer.fr (A.-A.S.); camille.roukoz@lyon.unicancer.fr (C.R.); 2 Radiation Therapy Unit, Azienda USL-IRCCS di Reggio Emilia, 42123 Reggio Emilia, Italy; sebastiano.finocchighersi@ausl.re.it (S.F.G.); mariapaola.ruggieri@ausl.re.it (M.P.R.); 3 Pathology Unit, Azienda Sanitaria Locale, Ospedale San Giacomo, 15067 Novi Ligure, Italy; ftava@aslal.it (F.T.); ctaverna@aslal.it (C.T.); 4 Radiation Oncology Unit, San Luca Hospital, AUSL Toscana Nord Ovest, 55100 Lucca, Italy; 5 Department of Radiation Oncology, Shohadaye Haft-e-Tir Hospital, Iran University of Medical Science, Teheran 1997667665, Iran; najafi.mas@iums.ac.ir; 6 Brachytherapy Unit, Department of Radiation Oncology, Catalan Institute of Oncology, University of Barcelona, L’Hospitalet de Llobregat, 08908 Barcelona, Spain; cgutierrezm@iconcologia.net; 7 Radiation Oncology Unit, International University Hospital Cheikh Zaid, Rabat 10000, Morocco; aminalazrekrak@lyon.unicancer.fr; 8 Interdisciplinary Department of Medicine, Section of Radiology and Radiation Oncology, University of Bari “Aldo Moro”, 70124 Bari, Italy; angela.sardaro@uniba.it; 9 Department of Health Sciences, Università del Piemonte Orientale UNIUPO, 28100 Novara, Italy

**Keywords:** radiation-associated breast angiosarcoma, radiation-induced breast angiosarcoma, RIAS, RAS, angiosarcoma, breast angiosarcoma, breast, systematic review

## Abstract

Introduction: Considering the increasing number of conservative surgeries (quadrantectomies) for primary breast carcinoma, especially in the early stages, often followed by adjuvant radiotherapy, the incidence of radiation-associated angiosarcoma (RAS) is expected to rise in the coming decades, and it will represent a clinical and therapeutic challenge, as limited data are available due to the rarity and heterogeneity of the disease. Though the prognosis of these patients is poor, a number of clinical and pathological factors can be evaluated to better understand the course of RAS. The aim of this systematic review is to explore the available clinical-pathological, therapeutic, and prognostic data regarding RAS to evaluate its occurrence, diagnosis, treatment, and outcomes. Method: RAS clinical data were identified by a systematic review conducted in five different databases (Medline, Web of Knowledge, Google Scholar, Scopus, and Cochrane). Only RAS works published in English, with access to the full manuscript text, and with clear results, were considered as eligible. Results: We considered 52 papers comprising 319 RAS cases. The patient age at diagnosis ranged from 37 to 93 years, with most cases occurring from 5 to 10 years after breast irradiation. The most common clinical presentation was an aggressive development of macules, papules, or erythematous-violaceous skin discoloration at the site of previous radiation treatment for primary breast carcinoma. Complete surgical excision appeared to be the first-line treatment of the disease, in some cases followed by adjuvant local and/or systemic therapies. Despite different treatments, local recurrence rates ranged from 40% to 90%, leading to negative outcomes and poor prognosis for patients. Conclusion: Although the literature is limited and the data are heterogeneous and contentious, our review aims to highlight the importance of early diagnosis, multimodal treatment, and long-term follow-up of RAS in order to limit and prevent the aggressiveness of this neoplasm.

## 1. Introduction

Breast carcinoma is the most common type of cancer in women and the second most frequent cancer-related cause of death [1]. Adjuvant radiotherapy (RT) has become increasingly important over the years, as conservative surgery for early-stage breast cancer has been demonstrated to result in progression-free survival (PFS) and overall survival (OS) rates comparable to radical mastectomy alone [2,3,4,5,6,7,8,9,10]. Indeed, over 60% of patients with breast cancer receive adjuvant RT, equating to over 10,000 women [11]. Moreover, RT may also represent a valid alternative to mastectomy in cases of breast carcinomas and as a salvage treatment for recurrences after initial conservative surgery [12].

Considering the high number of patients with breast cancer who undergo RT, it is mandatory to take into consideration the risk of developing radiation-induced tumors, as the association between ionizing radiation (IR) exposure and second malignancies has been well demonstrated in epidemiologic studies [13].

Breast angiosarcoma is a rare, aggressive disease composed of neoplastic endothelial cells. Two forms of the tumor are recognized: primary and radiation-associated angiosarcoma [14]. Radiation-associated breast angiosarcoma (RAS) occurs in oncological patients with a history of RT [15]. Although radiation is the main cause of RAS, some authors suggest that even the type of primary surgery can affect the risk of development of breast angiosarcoma, as cases treated with partial mastectomy have a higher (sevenfold) risk of developing this lesion compared to those treated with total mastectomy, but the data are conflicted. In the majority of cases, RT is administered as part of the primary treatment for breast carcinoma after breast surgery, though in sporadic cases it occurs after radiation treatment for other diseases, such as Hodgkin’s lymphoma [14,15,16,17,18].

Literature data report that RAS could affect up to 0.1% of all irradiated patients, with a median occurrence of 8 years from the end of RT (latency ranging from 12 to 291 months), with a mean age at diagnosis of 70 years [14]. Although it represents fewer than 1% of all breast cancers, RAS is characterized by an aggressive course and poor prognosis, with a 5-year OS around 20% due to the high rate of local recurrence and metastatic spread [15].

## 2. Angiosarcoma: Histopathology of a Rare and Insidious Disease

### 2.1. Pathological Features

In the fourth edition of the World Health Organization (WHO) classification of breast tumors [16], RAS is described separately from the primary form, as it is a unique entity, with specific morphological, cytological, and molecular features.

Macroscopically, the tumor has a hemorrhagic appearance and a friable consistency. The mass can be firm or spongy, with different degrees of cystic degeneration, blood-filled cysts, and cavities. On microscopic examination, most cases show dermal-based proliferation, with frank invasion of subcutaneous tissue and, in cases of partial mastectomy, of residual breast parenchyma.

**Figure 1:**
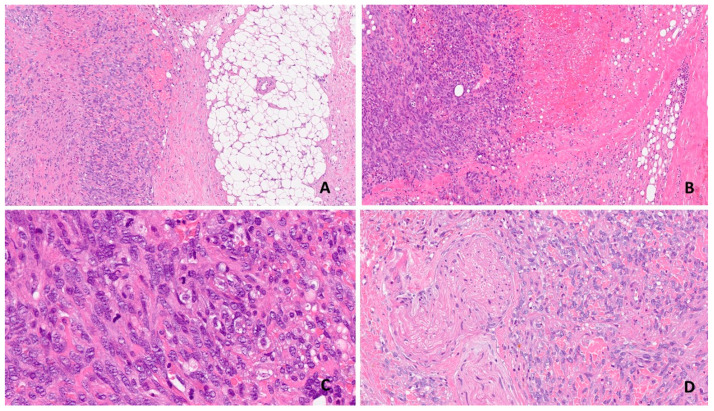
RAS. The lesion shows an infiltrative growth pattern with foci of neoplastic cells infiltrating the breast parenchyma and fat tissue (**A**); the cellulate component is interspersed in blood-filled spaces (**B**); abundant mitotic figures are present (**C**); the detail shows massive perineural invasion (**D**).

The majority of RAS tumors are high grade and poorly differentiated, in contrast to the primary counterpart, which more frequently shows a low or intermediate grade of differentiation. The lesion is usually multifocal or diffuse, with a variable degree of vasoformative or solid pattern of growth. It is composed of vascular elements with epithelioid to spindle morphology arranged in solid nests and short fascicles intermixed with blood-filled, slit-like spaces [17,18]. In the background, extravasate blood cells and hemorrhagic areas are typically seen, and the cellulate part can be found at the periphery of the lesion; hence, extensive sampling of prominent hemorrhagic lesions is mandatory. Cells show nuclei with vesicular chromatin and one or multiple prominent nucleoli. Mitoses are abundant, with typical and atypical figures (Figure 1).

In a minor proportion of cases, low- and intermediate-grade lesions are found, with less undifferentiated architecture. In low-grade forms, a vasoformative pattern of growth is prominent, with the presence of dilated and irregular neoplastic vessels lined by a single layer of neoplastic endothelial elements. Intermediate-grade neoplasms are characterized by neoplastic endothelial cells in multiple layers, with increased cytological atypia. A peculiar cytological feature of post-irradiation angiosarcoma of the breast is that cells exhibit high-grade features despite the general architectural grade and the amount of vessel formation. Occasional variants of RAS include tumors with a storiform pattern of growth, prominent spindle cell tumors, or the presence of cavernous elements. Thus, angiosarcomas that mimic metastatic breast carcinomas with epidermotropism can be found, leading to difficulties in differential diagnosis.

**Figure 2:**
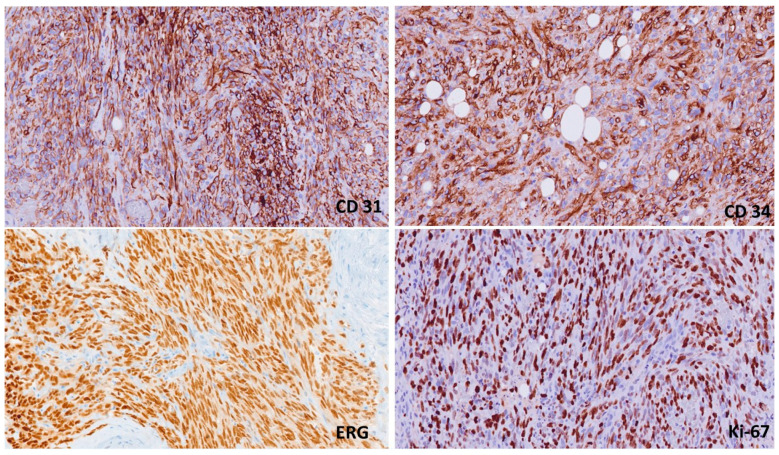
The immunohistochemical profile demonstrates positive staining for endothelial markers (CD31, CD34, ERG) and a high proliferation index (Ki-67: 85% of neoplastic cells).

Immunohistochemistry is useful in the diagnostic process, as the solid parts can resemble carcinomas or other types of sarcomas. Vimentin and FLI1 are usually positive, as are the endothelial markers CD31, ERG, CD34, and factor VIII. In a minority of cases, positivity for cytokeratin is found [19,20]. The proliferation index (MIB-1/Ki-67) is usually high. Estrogen and progesterone receptors are usually negative (Figure 2) [20,21].

Currently, different molecular pathways are involved in post-radiation angiosarcoma. Studies on the role of p53 loss of function and murine double minute 2 (MDM2) have shown that these two genes are occasionally mutated and/or downregulated. It has been shown that transgenic p53−/− mice have a high incidence of angiosarcoma (70%) [22]. MYC is a multifunctional, nuclear phosphoprotein that plays a role in cell cycle progression, apoptosis, and cellular transformation, and it stimulates angiogenesis and promotes metastasis. It was one of the first proto-oncogenes to be described and is deregulated in most tumor types. MYC high-level gene amplifications were observed in all secondary angiosarcoma cases but not in primary ones, suggesting that, despite their identical morphology, secondary angiosarcomas are genetically different from primary ones [20,21,22,23,24]. Guo et al. in 2011 hypothesized that Fms-related tyrosine kinase 4 (FLT4) encoding a tyrosine kinase receptor for vascular endothelial growth factors involved in lymphangiogenesis may be a potential candidate for this gene amplification [25]. A high-level gene amplification pattern was detected in 25% of radiation-induced angiosarcomas and in one post-lymphedema angiosarcoma analyzed and again co-amplified with MYC. This result suggests that FLT4 over-expression may represent a second step to progression of secondary angiosarcomas. All these findings suggest that MYC can be considered a hallmark of secondary angiosarcoma and may have implications for both the diagnosis and treatment of these tumors [26,27,28,29]. mTOR has been found to play a major role in cancer progression by acting as a master switch for cellular catabolism and anabolism, and consequently cell growth and proliferation and induction of cell cycle progression [29,30].

### 2.2. Differential Diagnosis

The main differential diagnosis is with primary angiosarcoma, mostly based on clinical and anamnestic data, even though some histological features can help in reaching the correct diagnosis, as shown in Table 1. Primary angiosarcoma usually occurs in deep breast parenchyma and displays a wider spectrum of histological appearance, and many cases display a low or intermediate grade. Depending on the grade, cells can be bland-looking, with minimal atypia and organized in a single layer with flat appearance (low-grade lesions) and up to progressive multilayering with a pseudopapillary pattern (moderate differentiated tumors). In high-grade angiosarcomas, the histological appearance is similar to the post-irradiation counterpart. Epithelioid angiosarcomas have a solid growth pattern, leading to difficulties in differential diagnosis with both RAS and carcinoma. Immunohistochemistry shows the same expression of vascular markers, with some lesions being positive for cytokeratins. Differently from RAS, the primary neoplasms lack MYC protein expression and MYC amplification.

Regarding other vascular lesions mimicking RAS, the most difficult differentiation is with the spectrum of atypical vascular lesions (AVLs). These lesions usually arise in the papillary and reticular dermis and are classified into lymphatic and vascular. AVLs and angiosarcomas have some overlapping features, such as the presence of at least a few focal areas of anastomotic vessels and hyperchromatic endothelial cells. Conversely, some diagnostic criteria belong mainly to angiosarcomas while they are absent in AVL, i.e., the infiltrative growth pattern with dissection of dermal collagen fibers and wide extension of the lesion into subcutaneous tissue, the associated papillary endothelial hyperplasia, and, primarily, the cytological prominent atypia of neoplastic cells, with marked pleomorphism and the presence of a large number of typical and atypical mitotic figures.

Moreover, if molecular tools are used, MYC amplification is typically found in RAS [18,31,32].

Other vascular lesions that can be found in breast skin or subcutis are different variants of hemangiomas and angiolipoma. Perilobular hemangioma is usually an incidental finding, with benign histological features composed of bland endothelial cells arranged in a lobulated architecture without anastomotic areas, and usually small. Other types of hemangiomas are found in the breast, such as the venous and the epithelioid variants, with similar features. In challenging cases or small biopsies, Ki-67 can be performed, showing low expression in hemangiomas and confirming their benign nature. In some cases, angiolipoma can be included in the differential diagnosis with angiosarcoma for the slit-like appearance of the vessels and the occasional spindle morphology of endothelial cells. The contextual presence of adipose tissue and the demarcation of the lesions, together with the absence of worrisome cytological features, usually lead to the correct diagnosis. Intravascular papillary endothelial hyperplasia (Masson tumor) is a benign, well-circumscribed, small-sized lesion, located in the subcutis of the breast. The center of the lesion is usually collagenized and sclerotic, while the typical papillary proliferation of a single layer of bland endothelial cells is present in the periphery, anastomotic vessels, cytological atypia, and unfitting borders are absent [33,34,35].

Metaplastic squamous cell carcinoma of the breast, especially the acantholytic pattern, can also be misdiagnosed as angiosarcoma. The epithelial cells, with similar grade of atypia and pleomorphism to those found in post-irradiated angiosarcoma, are arranged in loosely cohesive nests, forming pseudo-luminal structures. Immunohistochemistry including a wide panel of endothelial markers is mandatory, as cytokeratins are potentially expressed in both neoplasms [36,37,38]. Table 1 summarizes the main pathological characteristics of RAS compared to the primitive form.

### 2.3. Diagnosis

The diagnostic work-up of RAS includes imaging (mammography, ultrasound, or magnetic resonance imaging [MRI]), but biopsy is mandatory for certain diagnosis. Mammography features are often absent or nonspecific [39]. On ultrasound, RAS presents as a hypervascular and heterogeneous lesion with mixed hyper- and hypoechoic areas, with disruption of parenchymal structures [39]. Compared to mammography and ultrasound, MRI provides superior morphologic characterization and shows a heterogeneous mass with low T1 and high T2 signals, presumably due to the vascular origin of the tumor [40,41,42].

### 2.4. Rationale and Purpose of the Systematic Review

Currently, the standard treatment for RAS has not yet been identified, so mastectomy is recommended as the primary option [43]. Due to the progressive increase of breast cancer patients’ survival and the widespread adoption of adjuvant irradiation after conservative breast surgery, the incidence and prevalence of RAS are expected to rise in the coming decades, and, consequently, RAS management will be an emerging issue in the future of oncology [44]. The aim of the present systematic review is to investigate the current clinical experience regarding RAS, mainly exploring the treatments and clinical outcomes.

## 3. Materials and Methods

### 3.1. Search Strategy

The present systematic review followed the guidelines for the Preferred Reporting Items for Systematic Review and Meta-Analysis (PRISMA) revised in 2015 [45]. Databases including Medline, Web of Knowledge, Google Scholar, Scopus, and Cochrane were searched by two blinded investigators for all eligible studies based on works from 1987, the year when breast angiosarcoma was first described and published, up to September 2022.

Papers were considered that included selected words such as “breast angiosarcoma”, “secondary breast angiosarcoma”, “radiation-induced breast angiosarcoma”, “radiation-induced sarcoma of the breast”, “sarcoma and breast”, “angiosarcoma and breast”, “radiation therapy and sarcoma”, “radiation therapy and angiosarcoma”, “radiation therapy and breast sarcoma”, “post-irradiation angiosarcoma”, and “radiation-associated angiosarcoma”. The research involved an analysis of all studies published up to September 2022.

### 3.2. Selection Criteria

The inclusion criteria were as follows: (1) retrospective and prospective clinical trials, case reports or case series; (2) studies published in English. The exclusion criteria we used to better define the work herein described were as follows: (1) lack of access to the full text of the manuscript; (2) studies with incomplete data on clinical and pathological features, types of treatments, and follow-up and patient outcomes; (3) review papers; (4) studies not in English; (5) studies with patients not treated with radiotherapy who developed breast angiosarcomas; (6) studies with other types of radiation-induced sarcomas; (7) radiation-induced angiosarcomas not arisen in breast tissue.

### 3.3. Data Extraction

Two unblinded reviewers independently performed the data collection using struc-tured collection forms. We resolved disagreements by consensus or by involving a third person. The extracted data consisted of authors, year of publication, study design, major endpoints, sample size, age, stage, primary treatment, radiation treatment characteristics (technique, dose), RT dose, clinical presentation, time between RT and angiosarcoma manifestation, oncological treatments, median follow-up, and clinical outcomes.

### 3.4. Quality Assessment

The quality of the included articles was assessed by ascertaining a clear description of RAS onset time from radiotherapy, therapies used to treat the disease, and patient outcomes. Moreover, we used the Quality Assessment of Diagnostic Accuracy Studies 2 (QUADAS-2) tool.

## 4. Results

**Figure 3:**
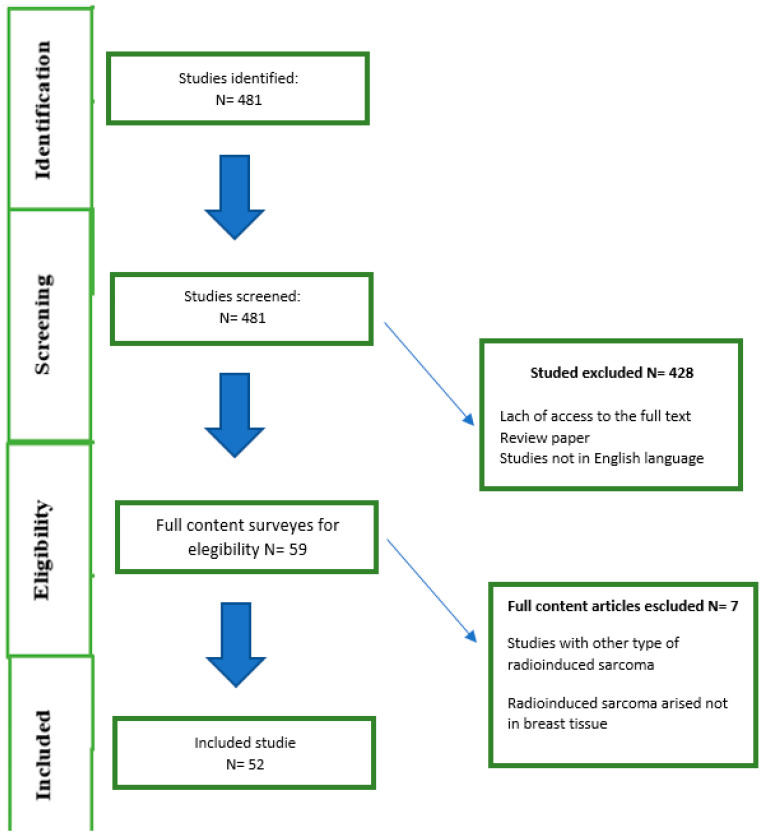
Flowchart for screening the eligible studies.

According to the purpose of this study, 481 articles were identified from the initial search. Subsequently, the 481 studies were reviewed by title and abstract. In the second step, the full texts of 54 studies were reviewed. Finally, 52 studies were included in our analysis (Figure 3), with a total number of 319 patients. Figure 1 shows the flowchart for screening the eligible studies.

All 52 selected studies were retrospective, and the majority were case reports. They were published between 2002 and 2021 (Table 2). All patients received conservative surgical treatment (lumpectomy) for the primary breast tumor, followed by adjuvant RT and hormone therapy. Systemic treatment was added in cases of advanced disease. In most cases, the entire breast volume was the target of RT, using conventional fractionation (1.8–2 Gy/day) or moderate hypofractionation (2.5–2.7 Gy/day), for a total of 45–60 Gy. A sequential boost of 5–10 Gy was also administered to the surgical bed in 18 patients. It should be noted that 4 patients did not receive RT on the entire breast volume, but only to the surgical bed according to the accelerated partial breast irradiation (APBI) technique [46,47,48].

### 4.1. Age and Clinical Presentation of RAS

The age of onset of RAS ranged from 37 to 88 years. Most of the patients were between 60 and 75 years of age. Only four patients were younger than 50 years. The clinical presentation included: macules, papules, alterations in the color of the skin, rash, nodules, ulceration, erythema, and skin thickening with lymphedema. In a few cases the clinical picture was more severe, with fibrous plaques, bleeding, and peau d’orange. In only one case the diagnosis was made by ultrasound examination conducted during the routine follow-up, as the patient did not show any clinical signs [82]. It should be noted that in most cases there was rapid growth of lesions with a very aggressive manifestation and spread of RAS. Of note, Tsapralis et al. [75] reported the first and only case of RAS following RT for male breast cancer.

Smith et al. [91] also reported cases with macroscopic extensive disease, involving the skin of the back, the umbelicus, clavicle, and chest wall. In two cases of the series, angiosarcoma was found after bioptical sample of suspect axillary lymph nodes.

### 4.2. Time between Primary Treatment and Onset of RAS

The time between RT for the primary breast tumor and the onset of RAS ranged from 1.5 to 19 years. Most patients developed radiation-associated cancer from 5 to 10 years after RT. Seven of 333 females developed RAS before the fifth year of follow-up after RT, and for 15 patients RAS occurred after the tenth year of follow-up.

### 4.3. RAS Treatments

The authors reported different treatments for RAS, involving single or combined approaches. Four patients underwent biopsy or lesion resection, one of whom subsequently underwent mastectomy for local recurrence.

Exclusive total mastectomy was the treatment of choice in one-third of the patients. Neoadjuvant chemotherapy was administered before mastectomy or in an adjuvant setting, and chemotherapy alone was administered in 35% of cases.

Exclusive chemotherapy was performed in 3.7% of patients. Finally, seven patients (2.3%) did not receive active treatments but only best supportive care. The main chemotherapy drugs used were anthracycline, doxorubicin, docetaxel, gemcitabine, epirubicin, and paclitaxel. A chemoimmunotherapy combination (nivolumab-paclitaxel) in a neoadjuvant setting was proposed in a few cases. Eight patients (2.7%) underwent a second course of RT (re-irradiation), which was occasionally combined with systemic therapy. Unfortunately, the re-irradiation doses were not always specified and mainly described as a generic “palliative radiotherapy”. By contrast, Uryvaev et al. [63] reported a retreatment on the chest wall with a dose of 50 Gy with conventional fractionation.

Two authors reported the use of hyperfractionated and accelerated re-irradiation (HART) in a small series of patients as neoadjuvant treatment or an adjuvant radiotherapeutic strategy for RAS [27,92].

### 4.4. Outcomes

The median follow-up ranged from 4 to 96 months. In six studies, no follow-up was reported. Instead, in 88 patients, there was no disease relapse, and therefore no evidence of disease was recorded at the end of the follow-up period. In the remaining cases, the patients developed a locoregional recurrence, either in the contralateral breast or distant metastasis. Considering the articles with 10 or more patients, the outcomes reported are described below:

- Amajoud et al. [71], with a follow-up ranging from 8 to 96 months, described 6 patients who developed early local recurrence and 2 patients who developed distant metastasis.
- Alves et al. [69] recorded a 5-year survival rate of 45%, with a follow-up of 9 years. Eleven patients died in the first 5 years after the diagnosis of RAS.
- Cohen et al. [68] reported a 2-year locoregional-free survival (LRFS) of 51.2% and 2-year distant metastasis-free survival (DMFS) of 67.3%.
- Mergancová et al. [44] reported 21 patients (40%) with no evidence of recurrence, while recurrence was reported in 23 out of 53 (43%). In 9 patients (17%), distant metastases were found. Regarding the survival rate, 43 patients (56%) at 3 years and 33 patients (33%) at 5 years were alive.
- Jallali et al. [56] found a significant relationship between incomplete surgical excision and patient outcomes, as when complete excision of the lesion was performed, the overall 2- and 5-year survival rates were 42% and 10%, respectively, while in patients with insufficient safe surgical margins, the 2- and 5-year overall survival rates were 0%.
- Of the 95 patients described by Torres et al. [57], 30 died of the disease and 17 patients died of other causes. The 1-, 2-, and 5-year OS were 91%, 78%, and 54%, respectively. The 1-, 2-, and 5-year DSS were 94%, 84%, and 63%. Patients who developed metastatic disease or local recurrence had significantly worse DSS rates than those who did not (*p* = 0.0002; Figure 1A); patients who presented initially with locally advanced disease or metastasis had worse DSS rates than patients who presented with localized primary tumors (*p* = 0.01; Figure 1B). The median survival time for patients with localized tumors was 7.3 (range, 0.2–15.8) years versus 4.7 (range, 0.7–10.1) years for those presenting with locally advanced disease. They reported a 5-year disease-specific survival (DSS) of 62.6%.
- Smith et al. [91] reported that 5 patients developed progressive disease in areas that were not covered by radiological treatment. One patient developed contralateral nodal disease, with complete regression after second HART followed by axillary dissection. One patient developed a chest recurrence and died of disease, and 3 patients died of metastatic disease with pulmonary and mediastinal localizations. In this series, the use of HART was reported to achieve full disease control in 10 of the 14 patients (71.4%). Regarding overall survival rates, 5 and 10 years were 79% and 63%, respectively, while 5 and 10 progression-free survival rates were both 64%. After a follow up of 8.1 years, 8 out of 14 (57.1%) patients were alive and free of disease, 2 (14.2%) died from intercurrent disease, and 4 (28.5%) died of angiosarcoma.

### 4.5. Quality Assessment

The included studies showed a significant heterogeneity of collected data. One study did not report patient age [87] and another the onset time of RAS after RT [56]. All authors reported RAS treatments except for Andrews et al. [54]. Six studies did not describe the duration of follow-up or the disease control [46,58,62,68,70,72]. Lastly, Miyata did not report the disease restaging at the last follow-up [79].

## 5. Discussion

In this paper, we aim to assess the state of the art of post-radiation breast angiosarcoma, a rare, long-term complication of breast carcinoma treated with conservative surgery followed by radiotherapy.

Radiotherapy represents a milestone treatment in many oncological pathologies [90,93,94,95,96] and is generally characterized by moderate toxicity. The most dreaded and rare late side effect is the onset of radiation-associated tumors. Among all types of radiation-associated sarcomas, angiosarcoma corresponds to approximately 40% [24,66,97].

Despite the well-defined origin and pathogenesis of this lesion and the advances in radiotherapy, a consensus of the best treatment for this disease has not yet been achieved, probably due to the rarity of the lesion, the wide age range of onset, and the different experience of oncologists and radiotherapists in different hospitals.

Pathogenesis: Several mechanisms have been proposed to explain the pathogenesis of RAS development. One hypothesis is that photons emitted during radiotherapy directly affect the DNA structure by inducing DNA breaks, particularly double-strand breaks. Another is related to the generation of reactive oxygen species that oxidize proteins and lipids but also induce additional damage to DNA, like the creation of basic sites and single-strand breaks. All these factors may cause genomic instability and promote mutations in several cancer-related genes. Regarding RAS, several gene mutations have been reported in the literature, such as the inactivation of the tumor suppressor gene p53 and the amplification of the 8q24 region containing the MYC oncogene.

Moreover, the prolonged cellular ischemic damage caused by radiation can contribute to inducing chronic lymphedema. In this situation, the increased levels of vascular growth factors within the tumor microenvironment can promote tumorigenesis, triggered by *FLT4* amplification (encoding VEGFR3) and *KDR* mutation (encoding VEGFR2). Finally, an association between breast cancer-related tumor-suppressor genes BRCA1/BRCA2 and RAS may also exist, although the exact mechanism has yet to be established [24,25,98].

Clinical data: Radiation-induced breast angiosarcoma has been more frequently found in elderly women with a history of breast conservation surgery and RT, since breast carcinoma commonly occurs in women between the ages of 55 and 70 years. After conservative breast surgery, angiosarcoma occurs in 0.005–1.11% of patients, with latency ranging from 1.5 to 18 years, often shorter compared to other types of sarcomas, with most patients developing the neoplasm 6 years after RT treatment [36,99].

Knowledge of the clinical presentation of RAS represents a key element for all physicians. The most common RAS manifestations reported are violaceous, red to purple macules, plaques, and nodules with or without skin thickening and breast swelling. Typically, these lesions rapidly converge and evolve into larger areas and nodules, often with ulceration in confirmed disease. In rare cases, no clinical signs are evident, and the neoplasm is detected with ultrasound [82].

Treatments: Surgical excision remains the main treatment for RAS with complete excision of the lesion, which often corresponds to total mastectomy, in some cases involving the thoracic wall. Tumor-free margins represent an important prognostic factor, as for some authors the status of the margins has more prognostic value than the surgical technique used [63]. However, no consensus is reported on safety centimeters, as most of the literature reports R0 margins without specifying the distance of the neoplasm from the margin. Some authors suggest that a 1-cm margin is recommended for small lesions, while for massive, highly infiltrative neoplasms, up to 3-cm margins are required [100,101]. One study proposed that in patients who receive adjuvant chemotherapy, 5-mm free margins are sufficient to help in decreasing the local recurrence rate and to improve overall survival [14].

Systemic therapy: Chemotherapy is frequently used in combination with surgery to optimize treatment and to achieve better overall survival and disease-free survival. Anthracycline, doxorubicin, docetaxel, gemcitabine, epirubicin, and paclitaxel were used in a number of studies. Systemic treatment can also be used as a single therapeutic option if surgery cannot be performed [81,82,86,102]. As immunotherapy is used in a wide spectrum of neoplasms with various degrees of response, Tammy Ju et al. reported a case of RAS treated with neoadjuvant immunotherapy in combination with chemotherapy (nivolumab-paclitaxel). The patient underwent mastectomy with complete pathological response [84].

To date, there is no established clear evidence on the best treatment because most data derive from case reports or case series. Aggressive surgical excision appears to be the most prevalent approach, with the majority of authors reporting mastectomy as the surgical option. Notwithstanding this, there are many experiences with nodulectomy, local excisions, or lumpectomy, without axillary dissection. For instance, Farran et al. [67] suggest that the presence of tumor-free margins after surgery is more important than the surgical technique itself for local disease control. A few papers report the use of a perioperative systemic therapy or an exclusive combined chemotherapy. Albeit isolated, the work of Ju et al. is of interest, where a multimodal strategy including preoperative chemo-immunotherapy followed by a mastectomy achieved a complete pathological response [81].

Despite the different treatments, the local recurrence rates remain very high, ranging from 40 to 90%, and many patients develop disease progression within months of starting chemotherapy. In many studies it is not possible to determine the long-term outcomes in view of the short follow-up period. As for systemic treatments, a large variety of therapeutic drugs are used; however, the taxanes and the anthracyclines appear to be the drugs with the highest tumor control probability. Considering the growing interest around the new target therapies for RAS management—namely, the anti-VEGF antibodies and the tyrosine kinase inhibitors, it is worth underlining that available data are scarce and new studies are mandatory.

The most controversial aspect of the treatment of RAS is the use of RT. Since RT is the cause of the onset of RIAS, it would be logical to avoid proposing it to patients with RAS. However, several studies have reported the use of re-irradiation in the adjuvant or neoadjuvant setting of RIAS/RAS [27,28,29,89,92,103,104]. A large study analyzed the role of adjuvant RT in RAS [105]. Interestingly, 17% of the patients who received re-irradiation had an increased 5-year local recurrence-free survival (LRFS) compared to those who did not undergo adjuvant RT (57% versus 34%, respectively). The patient selection criteria for adjuvant RT are heterogeneous and are often different for each center, depending on clinical practice and experience [30]. Modesto et al. reported a trend in OS benefit in patients who received adjuvant RT for RT-associated sarcomas in general [106], and an improved prognosis was demonstrated by two studies that included both primary and secondary tumors with adjuvant RT in breast angiosarcomas in general [31,107,108,109].

Hyperfractionated accelerated RT (HART) may be of particular benefit to RAS [34,35,91]. It has been evaluated as neoadjuvant or adjuvant therapy for secondary angiosarcomas. The adopted schedule with small tri-daily doses and the moderate total dose have a radiobiological rationale due to the high mitotic rate of RAS. The schedule consisted of three RT treatments per day, with a dose of 1 Gy per fraction, and a varying total dose depending on the risk for subclinical disease. The 5-year OS amounted to 86%, with an acceptable toxicity.

Although it is extremely difficult to summarize and no consensus is available, some advice for managing the RAS could be the following:

1. In consideration of the slow appearance of RAS, careful follow-up with clinical evaluation should be performed for a long time in patients treated for breast cancer so as to guarantee an early diagnosis.
2. A surgical biopsy must be able to reach a diagnosis of certainty.
3. The treatments must be planned by a multidisciplinary team.
4. The standard surgical procedure is mastectomy with negative margins. It is recommended that this should be carried out in a sarcoma specialist unit, especially if the tumor is beyond the confines of the remaining breast tissue or encroaching the chest wall.
5. However, in case of rapid growth after clinical manifestation, the tumor could be inoperable. For locally advanced inoperable or metastatic disease, chemotherapy is the pillar of treatment. Grade and surgical margin status are also important prognostic determinants in cases of post-irradiation sarcomas.
6. A definitive histological examination is also important in defining the prognosis of this disease. In fact, a higher grade of tumor is related to a higher risk of death in the first three years after diagnosis.
7. Treatment should be included in management guidelines for other soft-tissue sarcomas, such as those published by the European Society for Medical Oncology (ESMO) and the National Comprehensive Cancer Network (NCCN). Angiosarcomas are particularly sensitive to taxanes and liposomal doxorubicin. Weekly paclitaxel or liposomal doxorubicin may be considered as valid alternatives to standard anthracyclines plus/minus ifosfamide treatment for this particular histology in view of their manageable adverse effect profile.
8. The possibility of re-irradiation in case of risk of local recurrence should be evaluated. Considering the high growth rate of breast angiosarcomas, the use of hyperfractionated radiotherapy could make the tumor cells more likely to repopulate between daily fractions of radiotherapy.

### Study Limitations

Our review has some limitations. Most of the included studies are case reports/series, and all of them have a retrospective design. The treatment types (surgery, chemotherapy, and RT) are very heterogeneous among the different studies. Moreover, the authors reported different possibilities for each type of treatment (different chemotherapeutic drugs, different surgery techniques, and different RT schedules). Lastly, the outcomes are not described at the same timepoints following the therapies, with various endpoints.

## 6. Conclusions

Radiation-associated breast angiosarcoma is a rare and aggressive type of cancer, which may occur as a secondary, late effect of breast irradiation after conservative breast cancer treatment. Despite the significant improvement of local and systemic therapy and surgery, RAS is still a matter of debate, and many aspects regarding the origin and management of the disease are controversial. Moreover, the rarity and lack of standardization in the approach to RAS contribute to highlighting the gray zones of this neoplasm.

However, the available data do postulate some conclusions. Patients who must undergo adjuvant breast radiotherapy need to be aware of the risk, albeit low, of RAS occurrence. On the other hand, all the specialists involved in patient follow-up must be very careful in searching for the early signs and symptoms of the disease. For all the reasons herein described, a multimodal approach is required in order to treat patients with a personalized and complete therapeutical approach.

Because of the rarity of RAS worldwide, prospective studies are not conceivable. Therefore, retrospective data collection with multicenter large international data sets is desirable in order to better understand the management and role of the different therapeutic strategies.

## Figures and Tables

**Table 1:** Main pathological characteristics of RAS compared to the primitive breast form.

	Primary/sporadic angiosarcoma			RAS
Site	Breast parenchyma			Breast dermis and subcutis; rare parenchymal involvement
Histological Features	Low-grade Type 1	Intermediate-grade Type 2	High-grade Type 3	Usually high-grade
Tumoral vessels	Large, dilated, anastomotic, well-formed	Dilated, angulated	Poorly formed	Irregular, angulated Slit-like morphology Poorly formed
Cellularity	Low	Intermediate	High	High
Papillary formation	Absent/Infrequent	Present	Prominent	Present/Prominent
Arrangement of neoplastic cells	Single layer	Multilayer, Hobnail	Multilayer, solid growth	Multilayer
Endothelial tufting	Minimal	Moderate	Prominent	Prominent
Blood extravasation/ Hemorrhage	Rare	Moderate	Present “Blood lake” formation	Present Prominent “Blood lake” formation
Solid/spindle cell foci	Absent	Minimal	Present Prominent	Prominent
Nuclei	Often incospiciuous Occasionally hyperchromatic and prominent	In cellular areas Hyperchromatic and pleomorphic	Diffusely and pleomorphic	Poorly differentiated nuclei Dark chromatin Prominent nucleoli
Mitosis	Occasional	Present in cellular areas	Present	Variable 1 to 35/10 HPF (Mean 9/10 HPF)
Necrosis	Absent	Absent	Present	Present
IHC				
Ki-67 (% of cells)	Low	Intermediate	High	High
Vimentin	Positive	Positive	Positive	Positive
CD34, CD31	Positive	Positive	Positive	Positive
D2-40, FLI-1, HIF1A	Variable	Variable	Variable	Variable
EMA, S-100, CD68, desmin HHV-8	Negative	Negative	Negative	Negative
ER, PR	Negative	Negative	Negative	Negative
Cytokeratin	Negative	Negative	Variable	Variable
c-MYC protein	Negative	Negative	Negative	Positive
Genetic Studies				
MYC	Wild type	Wild type	Wild type	Amplification

**Table 2:** Panel of the 52 selected studies.

Study	N of pts	Age (Mean)	Clinical Presentation	Time from RT	Treatments	Median Follow-Up	Outcome
De Bree et al. (2002) [49]	1	76	- bluish discoloration - bilateral lesion	10 and 12 yrs	Wide mastectomy—simple mastectomy	2 and 4 yrs	NED
Anania et al. (2002) [50]	1	46	- hard nodule appeared at the subcicatricial site	10 yrs	Simple mastectomy	4 yrs	Relapse and second surgery—NED
Seo et al. (2003) [51]	1	72	- areas of erythematous lesions - bluish discoloration	5 yrs	Mastectomy	N/A	N/A
Brenn T et al. (2005) [21]	26	62	- violaceous plaques - nodules - ecchymoses - small papules	6 yrs	Mastectomy/wide excision	1.5 yrs	NED (57%) Recurrence (42%)
Tomasello et al. (2006) [52]	1	83	- erythematous and violaceous nodular skin lesions rapidly growing	5 yrs	CHT (paclitaxel)	5 days	Deceased
Mano et al. (2006) [53]	1	71	- breast nodules	10 yrs	CHT + Mastectomy	4 mos	NED
Patton KT et al. (2008) [33]	1	61	- colored papules - erythematous papules/patches/plaques ranging in size from 1 to 60 mm	6 yrs	Surgery	47 mos	46% NED
Perez-Ruiz et al. (2009) [46]	1	65	- macular lesions	5 yrs	CHT (paclitaxel)	4 mos	Relapse
Andrews S et al. (2010) [54]	1	74	- multiple islands of violaceous macules, plaques, and nodules around the peripheral circumference of the high-dose irradiated field	4 yrs	N/A	N/A	N/A
Arnaout et al. (2012) [55]	1	73	- ecchymosis - skin thickening	8 yrs	CHT (gemcitabine-taxane) + mastectomy	1 mos	NED
Jallali et al. (2012) [56]	14	68	- purple - discoloration - eczematous rash - hematoma-like appearance - breast swelling	6.75 yrs	Surgical excision (complete and incomplete) Neoadjuvant chemotherapy	15 mos	Recurrence 12/14 (85%)
Torres KE et al. (2013) [57]	95	68	- nodules - skin changes - macular lesion - skin ulceration	7 yrs	Surgery ± CHT neoadjuvant or adjuvant	10.8 y	NED (51%) Recurrence (48%) DSS 62.6%
Colwick et al. (2013) [58]	1	77	- rash over lumpectomy scar	12 yrs	Mastectomy + RT	11 mos	Recurrence
Hoffmann et al. (2013) [47]	1	85	- a livid cuaneous nodule and bluish discoloration and a fibrotic transformation of the skin with livid hemorrhagic intrusions	12 yrs	Mastectomy + CHT (4 weeks later doxorubicin for local relapse, then paclitaxel)	6 mos	Deceased
Mansfield SA et al. (2014) [59]	1	75	- bruising to left breast	8 yrs	Surgery + HT	5 mos	NED
Zemanova et al. (2014) [60]	3	44	- skin inflammation, skin nodules, skin swelling	9.3 yrs	(1) CHT + mastectomy (2) Mastectomy (3) Mastectomy + RT	5 yrs	NED
Azzariti et al. (2014) [61]	1	71	- purple-blue painless skin lesion at the site of the surgical scar that evolved into - an ulcerated lesion of about 1.5 cm	9 yrs	Mastectomy	24 mos	Relapse
Tanaka et al. (2015) [62]	1	73	- palpable mass with reddening of the skin	18 yrs L breast 12 yrs R breast	Mastectomy	N/A	NED at the time of report
Uryvaev et al. (2015) [63]	6	78	- purple-blue cutaneous lesion	9.2 yrs	Mastectomy ± CRT	3.4 yrs	NED (66%) Recurrence (33%)
Tato-Varela et al. (2016) [64]	1	62	- multiple skin lesions	8 yrs	Bilateral mastectomy	1 yr	NED
Mocerino et al. (2016) [65]	1	77	- ecchymotic lesion (1.3 cm) near the scar of the previous breast surgery	8 yrs	Mastectomy	1 yr	Relapse
Peterson et al. (2016) [66]	1	72	- periareolar infiltrated erythematous papules - reddish nodules	14 yrs	Mastectomy	20 mos	NED
Farran Y et al. (2017) [67]	1	67	- circumscribed red skin discoloration	N/A	Surgery	5 mos	NED
Cohen-Hallaleh et al. (2017) [68]	49	72	- purple-blue cutaneous lesions	7.5 yrs	Mastectomy ± CHT	2 years	NED (48%) Local recurrence (51.4%)
Alves I et al. (2018) [69]	11	71.5	- skin lesions (7) - ski lesions associated to palpable mass (4)	8.9 yrs	Mastectomy ± CHT	5 yrs	5-year survival rate: 45%
García Novoab et al. (2018) [70]	1	37	- wine-red bump with a shiny halo	4 yrs	Surgery	N/A	N/A
Amajoud (2018) [71]	10	65	- thickening of skin, blue discoloration rash, nodules and small hematomas	7.3 yrs	Mastectomy	27.8	Local recurrence (70%) NED (30%)
Lyou Y et al. (2018) [72]	2	(1) 68 (2) 44	- multiple nodules of vascular proliferation throughout the dermis	(1) 6 yrs (2) 13 yrs	1. BCS 2. Surgery	N/A	(1) N/A (2) NED
Horisawa N et al. (2018) [73]	1	76	- mass occurring on the skin	6 yrs	Surgery + radiotherapy	3 yrs	NED
Verdura et al. (2019) [74]	1	79	- skin ulcers	8 yrs	CHT + mastectomy	12 mos	NED
Tsapralis et al. (2019) [75]	1	72 male	- nodules	6 yrs	Mastectomy + salvage surgery + CHT (paclitaxel, pazopanib)	32 mos	Deceased
Oliveira LAA et al. (2020) [76]	1	73	- two pinkish-violaceous lesions	7 yrs	Mastectomy	2 yrs	NED
Majdoubi A (2020) [77]	1	43	- 3 cm nodule - erythemato-violaceous macules	6 yrs	Mastectomy + CHT (anthracycline + cyclophosphamide)	6 mos	NED
Abbenante D et al. (2020) [78]	1	70	- numerous erythemato-violaceous macules - papules with the tendency to merge into plaques	14 yrs	Mastectomy	4 mos	NED
Miyata et al. (2020) [79]	1	88	- red skin, inflammation	8 yrs	RT + CHT	8 mos	NED
Shiraki et al. (2020) [80]	2	(1) 72; (2) 80	- (1) red skin inflammation - (2) skin nodules	(1) 5 yrs (2) 3 years	(1–2) Mastectomy	(1.2) 1 yr	(1–2) 1 year recurrence
Jayaraiah et al. (2020) [81]	1	62	- enlarging right breast lump - skin erosion - bleeding - small lesion of 0.5 cm on skin	5 yrs	Bilateral mastectomy + CHT	17 mos	NED
Lewcun et al. (2020) [82]	1	64	- skin nodules/skin thickening	6 yrs	CHT + Mastectomy	2 yrs	NED
Suzuki (2020) [83]	1	62	- absence of clinical signs	8 yrs	Mastectomy + CHT	8 mos	NED
Kacen et al. (2021) [84]	1	60	- erythematous skin discoloration - with multiple dermal lesions	3 yrs	CHT + mastectomy	6 mos	Relapse
Tammy Ju et al. (2021) [48]	1	85	- redness - black nodules	7 yrs	CHT-IT (nivolumab + paclitaxel) + Mastectomy	1 yr	NED
Javed et al. (2021) [85]	1	73	- rapid appearance of multiple evolving, nontender, violaceous patches (diameter: 7 cm)	12 yrs	Mastectomy + CHT (12 weeks-Taxol)	6 mos	NED
Cozzi et al. (2021) [86]	2	61, 69	- palpable nodule, - solid skin lesions	(1) 8 yrs (2) 8 yrs	(1) Mastectomy (2) Mastectomy + CHT	(1) 2 yrs (2) 6 mos	(1) NED (2) Relapse
Mergancová et al. (2022) [44]	53	72	- skin erosion - bleeding - rapidly ulcerating mass	6.5 yrs	(1) Mastectomy (2) Mastectomy + CHT (3) Mastectomy + RT	30 mos (mean)	NED (40%) Recurrence (43%) Progression (17%)
Jayarajah U et al. (2020) [81]	1	57	- rapidly growing skin nodule on right breast and small nodule on left breast	5 yrs	Mastectomy + CHT	15 mos	NED
Gambini et al. (2015) [87]	2		- skin nodules	(1) 5 yrs (2) 4.4 yrs	Mastectomy + CHT	4 yrs	(1) NED (2) Mild progression
Retter et al. (2022) [88]	1	77	- skin nodules	10 yrs	Mastectomy	2 yrs	NED
Horevoets et al. (2013) [89]	1	61	- erythematous skin lesion	19 yrs	Wide local excision	1.2 yrs	NED
Tahir et al. (2006) [90]	1	78	- nodules, peau d’orange, skin swelling	9 yrs	Mastectomy	1 yr	NED
Looi WS et al. (2022) [27]	6	73	- erythematous or violaceous skin - thickened, and ecchymotic skin	9.1 yrs	3 Pre-op HART + mastectomy 2 Mastectomy with local chest wall recurrence treated with definitive HART 1 Definitive HART	1.5 yrs	4 NED 2 Deceased
Smith T.L. et al. (2014) [91]	14	63	Local disease—peau d’orange, brawny, or ecchymotic skin changes, and intact or bleeding nodular or vesicular satellite nodules. Extensive macroscopic disease extended to the back, to the level of the umbilicus, to the clavicle, and chest wall Axillay lymph nodes	7.7 yrs	8 HART folllowed by mastectomy 5 Mastectomy followed by HART 1 Definitive HART	8.1 years	8 NED 6 Deceased

Abbreviations: N: number; PTS: patients; yrs: years; mos: months; N/A: not available; NED: no evidence of disease; RT: radiotherapy; CHT: chemotherapy; HT: hormone therapy; IT: immunotherapy; BCS: conservative breast surgery; DSS: disease specific survival; HART: hyperfractionated accelerated re-irradiation.

## Data Availability

Not applicable.
